# Label-free proteomic analysis to confirm the predicted proteome of *Corynebacterium pseudotuberculosis* under nitrosative stress mediated by nitric oxide

**DOI:** 10.1186/1471-2164-15-1065

**Published:** 2014-12-04

**Authors:** Wanderson M Silva, Rodrigo D Carvalho, Siomar C Soares, Isabela FS Bastos, Edson L Folador, Gustavo HMF Souza, Yves Le Loir, Anderson Miyoshi, Artur Silva, Vasco Azevedo

**Affiliations:** Depto de Biologia Geral, Instituto de Ciências Biológicas, Universidade Federal de Minas Gerais, Belo Horizonte, Brazil; Instituto de Ciências Biológicas, Universidade Federal do Pará, Belém, Pará Brazil; Waters Corporation, MS Applications and Development Laboratory, São Paulo, Brazil; Institut National de la Recherche Agronomique - INRA, UMR1253 STLO, Rennes, 35042 France; Agrocampus Ouest, UMR1253 STLO, Rennes, 35042 France

**Keywords:** *Corynebacterium pseudotuberculosis*, Caseous lymphadenitis, Proteomics, Label-free proteomics, Nitrosative stress, Nitric oxide

## Abstract

**Background:**

*Corynebacterium pseudotuberculosis* biovar *ovis* is a facultative intracellular pathogen, and the etiological agent of caseous lymphadenitis in small ruminants. During the infection process, the bacterium is subjected to several stress conditions, including nitrosative stress, which is caused by nitric oxide (NO). *In silico* analysis of the genome of *C. pseudotuberculosis ovis* 1002 predicted several genes that could influence the resistance of this pathogen to nitrosative stress. Here, we applied high-throughput proteomics using high definition mass spectrometry to characterize the functional genome of *C. pseudotuberculosis ovis* 1002 in the presence of NO-donor Diethylenetriamine/nitric oxide adduct (DETA/NO), with the aim of identifying proteins involved in nitrosative stress resistance.

**Results:**

We characterized 835 proteins, representing approximately 41% of the predicted proteome of *C. pseudotuberculosis ovis* 1002, following exposure to nitrosative stress. In total, 102 proteins were exclusive to the proteome of DETA/NO-induced cells, and a further 58 proteins were differentially regulated between the DETA/NO and control conditions. An interactomic analysis of the differential proteome of *C. pseudotuberculosis* in response to nitrosative stress was also performed. Our proteomic data set suggested the activation of both a general stress response and a specific nitrosative stress response, as well as changes in proteins involved in cellular metabolism, detoxification, transcriptional regulation, and DNA synthesis and repair.

**Conclusions:**

Our proteomic analysis validated previously-determined *in silico* data for *C. pseudotuberculosis ovis* 1002. In addition, proteomic screening performed in the presence of NO enabled the identification of a set of factors that can influence the resistance and survival of *C. pseudotuberculosis* during exposure to nitrosative stress.

**Electronic supplementary material:**

The online version of this article (doi:10.1186/1471-2164-15-1065) contains supplementary material, which is available to authorized users.

## Background

*Corynebacterium pseudotuberculosis* is a Gram-positive, facultative, intracellular pathogen belonging to the *Corynebacterium*, *Mycobacterium*, *Nocardia*, or CMN, group. This group belongs to the phylum Actinobacteria. The defining characteristics of the CMN group are a specific cell wall organization, consisting of peptidoglycan, arabinogalactan, and mycolic acids, and a high chromosomal G + C content [1]. *C. pseudotuberculosis ovis* is the etiological agent of the chronic infectious disease caseous lymphadenitis, which affects small ruminants worldwide. As a result, *C. pseudotuberculosis ovis* is responsible for significant economic losses in the goat and sheep industries, mainly stemming from decreased meat, wool, and milk production, reproductive disorders, and carcass contamination [1, 2]. Bacterial factors that contribute to the virulence of *C. pseudotuberculosis* include phospholipase D [3], toxic cell wall lipids [4], and the iron transporter *fagABC* complex [5].

*In silico* analysis of the genome of *C. pseudotuberculosis ovis* 1002 [6], as well as the pan-genome analysis of 15 other strains of *C. pseudotuberculosis*[7], identified genes involved in the response of this pathogen to different types of stress. Recently, the functional genome of *C. pseudotuberculosis ovis* 1002 was evaluated at the transcriptional level following exposure to different types of abiotic stress, including heat, osmotic, and acid stresses [8]. This allowed the characterization of several genes involved in distinct biological processes that favor the survival of the pathogen under the given stress condition.

However, during the infection process, *C. pseudotuberculosis* encounters nitrosative stress, caused by nitric oxide (NO), in the macrophage intracellular environment. A reactive nitrogen species (RNS) found in mammalian systems, NO is produced from L-arginine by NO synthases (NOS), and is present in three isoforms: endothelial NOS, neuronal NOS, involved in blood pressure control and neural signaling, and inducible NOS, associated with host defenses [9, 10]. The NO produced during bacterial infection has antimicrobial properties, killing pathogens by causing damage to DNA, RNA, and proteins [11]. However, several pathogens contain pathways that allow bacterial survival under nitrosative stress conditions, including NO-sensitive transcriptional regulators [12], DNA and protein repair systems [13], and antioxidant systems [14].

Currently, little is known about the factors involved in the resistance of *C. pseudotuberculosis* to nitrosative stress. Pacheco et al. [15] showed that the alternative sigma (σ) factor, σ^E^, plays a role in the survival of *C. pseudotuberculosis* in the presence of RNS. A σ^E^ null strain showed increased susceptibility to nitric oxide compared with the wild-type, and, in an *in vivo* assay, was unable to persist in mice. However, in iNOS-deficient mice, the mutant strain maintained its virulence [15]. In the same study, the extracellular proteome of *C. pseudotuberculosis* was analyzed in response to nitrosative stress, allowing the characterization of proteins that contribute to the adaptive processes of the pathogen in this environment [15].

To complement the results obtained in previous studies, and to identify factors involved in the survival of *C. pseudotuberculosis* under nitrosative stress conditions, we applied high-throughput proteomics using an liquid chromatograph high definition mass spectrometry (LC-HDMS^E^) (data-independent acquisition, in ion mobility mode) approach to evaluate the global expression of the functional genome of *C. pseudotuberculosis ovis* 1002 at the protein level under nitrosative stress conditions.

## Methods

### Bacterial strain and growth conditions

*C. pseudotuberculosis* biovar *ovis* strain 1002, isolated from a goat, was maintained in brain heart infusion broth (BHI; HiMedia Laboratories Pvt. Ltd., Mumbai, India) at 37°C. For stress-resistance assays, strain 1002 was cultivated in a chemically-defined medium (CDM), containing Na_2_HPO_4_.7H_2_O (12.93 g/l), KH_2_PO_4_ (2.55 g/l), NH_4_Cl (1 g/l), MgSO_4_.7H_2_O (0.20 g/l), CaCl_2_ (0.02 g/l), 0.05% (v/v) Tween 80, 4% (v/v) MEM vitamin solution (Invitrogen, Gaithersburg, MD, USA), 1% (v/v) MEM amino acid solution (Invitrogen), 1% (v/v) MEM non-essential amino acid solution (Invitrogen), and 1.2% (w/v) glucose, at 37°C [16].

### Nitric oxide assay and preparation of whole bacterial lysates

Diethylenetriamine/nitric oxide adduct (DETA/NO) resistance of *C. pseudotuberculosis* was characterized as previously described [15]. When strain 1002 reached exponential growth phase (OD_600_ = 0.6) in the chemically-defined medium, the culture was divided into two aliquots (control condition, strain 1002_Ct; NO exposure, strain 1002_*DETA/NO*), and DETA/NO was added to the appropriate aliquot to a final concentration of 0.5 mM. The growth of strain 1002 in the presence of DETA/NO was then evaluated for 10 h. For proteomic analysis, protein was extracted after 1 h of exposure to DETA/NO. Both the control and DETA/NO cultures were centrifuged at 4,000 × *g* for 10 min at 4°C. The cell pellets were washed in phosphate buffered saline and then resuspended in 1 ml of lysis buffer (7 M urea, 2 M thiourea, 4% (w/v) CHAPS, and 1 M dithiothreitol (DTT)). The cells were then sonicated using five 1-min cycles on ice. The resulting lysates were centrifuged at 14,000 × *g* for 30 min at 4°C. The extracted proteins were then submitted to centrifugation at 13,000 × *g* for 10 min using a spin column with a threshold of 10 kDa (Millipore, Billerica, USA). Proteins were denatured with (0.1% (w/v) *Rapi*GEST SF surfactant at 60°C for 15 min (Waters, Milford, CA, USA), reduced using 10 mM DTT for 30 min at 60°C, and alkylated with 10 mM iodoacetamide in a dark chamber at 25°C for 30 min. Next, the proteins were enzymatically digested with 1:50 (w/w) trypsin at 37°C for 16 hours (sequencing grade modified trypsin; Promega, Madison, WI, USA). The digestion process was stopped by adding 10 μl of 5% (v/v) Trifluoroacetic acid (TFA) (Fluka, Buchs, Germany). Glycogen phosphorylase was added to the digests to a final concentration of 20 fmol/μl as an internal standard for normalization prior to each replicate injection. Analysis was carried out using a two-dimensional reversed phase (2D RP-RP) nanoUPLC-MS (Nano Ultra Performance Liquid Chromatography) approach, using multiplexed HDMS^E^ label-free quantitation as described previously [17].

### LC-HDMS^E^ analysis and data processing

Qualitative and quantitative by 2D nanoUPLC tandem nanoESI-HDMS^E^ (Nano Electrospray High Definition Mass Spectrometry) experiments were conducted using a 1-h reversed phase (RP) acetonitrile (0.1% v/v formic acid) gradient (7–40% (v/v)) at 500 nl/min on a nanoACQUITY UPLC 2D RP × RP Technology system [18]. A nanoACQUITY UPLC High Strength Silica (HSS) T3 1.8 μm 75 μm × 15 cm column (pH 3) was used in conjunction with a RP XBridge BEH130 C18 5 μm 300 μm × 50 mm nanoflow column (pH 10). Typical on-column sample loads were 250 ng of the total protein digests for each of the five fractions (250 ng/fraction/load). For all measurements, the mass spectrometer was operated in resolution mode, with a typical effective *m/z* conjoined ion-mobility resolving power of at least 1.5 M FWHM, an ion mobility cell filled with nitrogen gas, and a cross-section resolving power at least 40 Ω/ΔΩ. All analyses were performed using nano-electrospray ionization in the positive ion mode nanoESI (+), and a NanoLockSpray (Waters) ionization source. The lock mass channel was sampled every 30 s. The mass spectrometer was calibrated with a MS/MS spectrum of [Glu^1^]-fibrinopeptide B (Glu-Fib) human solution (100 fmol/μl) delivered though the reference sprayer of the NanoLockSpray source. The double-charged ion ([M + 2H]^2+^ = 785.8426) was used for initial single-point calibration, and MS/MS fragment ions of Glu-Fib were used to obtain the final instrument calibration. Multiplexed data-independent scanning with added specificity and selectivity of a non-linear “T-wave” ion mobility (HDMS^E^) experiments were performed using a Synapt G2-S HDMS mass spectrometer (Waters). The mass spectrometer was set to switch automatically between standard MS (3 eV) and elevated collision energies HDMS^E^ (19–45 eV) applied to the transfer “T-wave” collision-induced dissociation cell with argon gas. The trap collision cell was adjusted for 1 eV using a millisecond scan time adjusted based on the linear velocity of the chromatography peak delivered though nanoACQUITY UPLC, to obtain a minimum of 20 scan points for each single peak at both low-energy and high-energy transmission, followed by an orthogonal acceleration time-of-flight from 50–2000 *m/z*. The radio frequency (RF) offset (MS profile) was adjusted so that the nanoUPLC-HDMS^E^ data were effectively acquired from an *m/z* range of 400–2000, which ensured that any masses observed in the high energy spectra of less than 400 *m/z* arose from dissociations in the collision cell.

### Data processing

Protein identification and quantitative data packaging were generated using dedicated algorithms [19, 20], and by searching against a *C. pseudotuberculosis* database with default parameters for ion accounting [21]. The databases were reversed “on-the fly” during the database query searches, and appended to the original database to assess the false positive rate of identification. For proper processing of spectra and database searching conditions, ProteinLynxGlobalServer v.2.5.2 (PLGS) with Identity^E^ and Expression^E^ informatics v.2.5.2 (Waters) were used. UniProtKB (release 2013_01) with manually-reviewed annotations was also used, and the search conditions were based on taxonomy (*C. pseudotuberculosis*), maximum missed cleavages by trypsin allowed up to one, and variable carbamidomethyl, acetyl N-terminal, phosphoryl, and oxidation (M) modifications [21, 22]. The Identity^E^ algorithm with Hi3 methodology was used for protein quantitation. The search threshold for accepting each individual spectrum was set to the default value, with a false-positive value of 4%. Biological variability was addressed by analyzing each culture three times. Normalization was performed using the Expression^E^ tool with a housekeeping protein that showed no significant difference in abundance across all injections. The proteins obtained were organized by the PLGS Expression^E^ tool algorithm into a statistically significant list corresponding to increased and decreased regulation ratios among the different groups. The quantitation values were averaged over all of the samples, and the quoted standard deviations at *p* ≤ 0.05 in the Expression^E^ software refer to the differences between biological replicates. Only proteins with a differential expression log_2_ ratio between the two conditions greater than or equal to 1.2 were considered [23].

### Bioinformatics analysis

The identified proteins were analyzed using the prediction tools SurfG+ v1.0 [24], to predict sub-cellular localization, and Blast2GO, to predict gene ontology functional annotations [25]. The PIPS software predicted proteins present in pathogenicity islands [26]. The protein-protein interaction network was constructed using interolog mapping methodology and metrics according to Rezende et al. [27]. A preview of the interaction network was generated using Cytoscape version 2.8.3 [28], with a spring-embedded layout. CMRegNet was used to predict gene regulatory networks [29].

## Results

### Effects of nitric oxide on the growth of *C. pseudotuberculosis*

**Figure 1:**
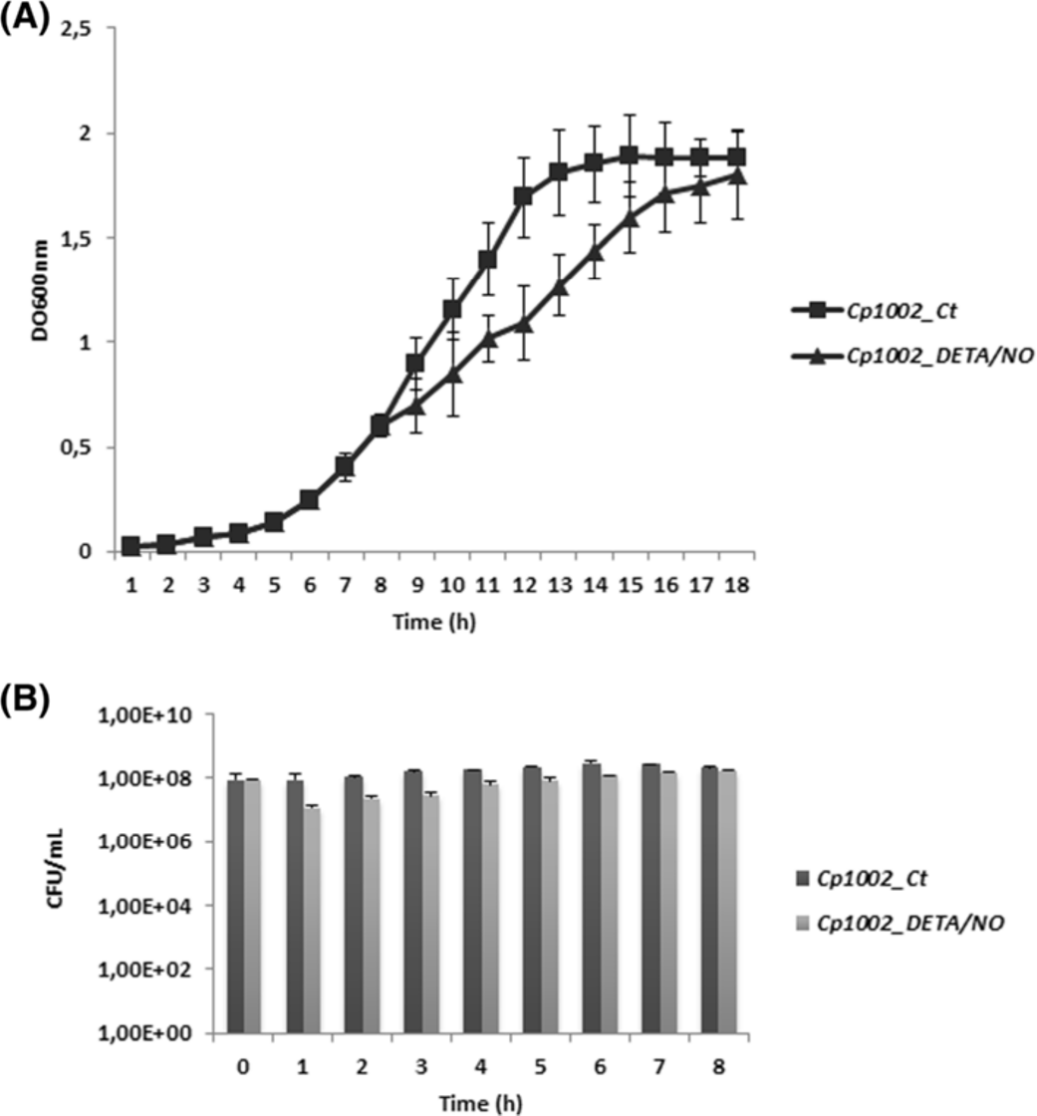
**Growth and survival profile of** ***C. pseudotuberculosis*** **during NO exposure. (A)** Growth of *C. pseudotuberculosis* after 10 h exposure to 0.5 mM DETA/NO. **(B)** Survival of *C. pseudotuberculosis* evaluated by colony forming units. The results shown in A and B represent an average of three independent experiments.

In this study, we examined the exponential growth of *C. pseudotuberculosis* strain 1002 under nitrosative stress. The growth and cell viability of strain 1002 was monitored for 10 h with and without DETA/NO supplementation (Figure 1). The control culture reached stationary phase by 5 h post-inoculation, while the culture containing DETA/NO did not reach stationary phase until approximately 10 h post-inoculation. However, these results showed that although DETA/NO (0.5 mM) affected the growth rate, *C. pseudotuberculosis* likely contains factors that promote survival in the presence of RNS.

### Label-free proteomic analysis of *C. pseudotuberculosis*grown under nitrosative stress conditions

**Figure 2:**
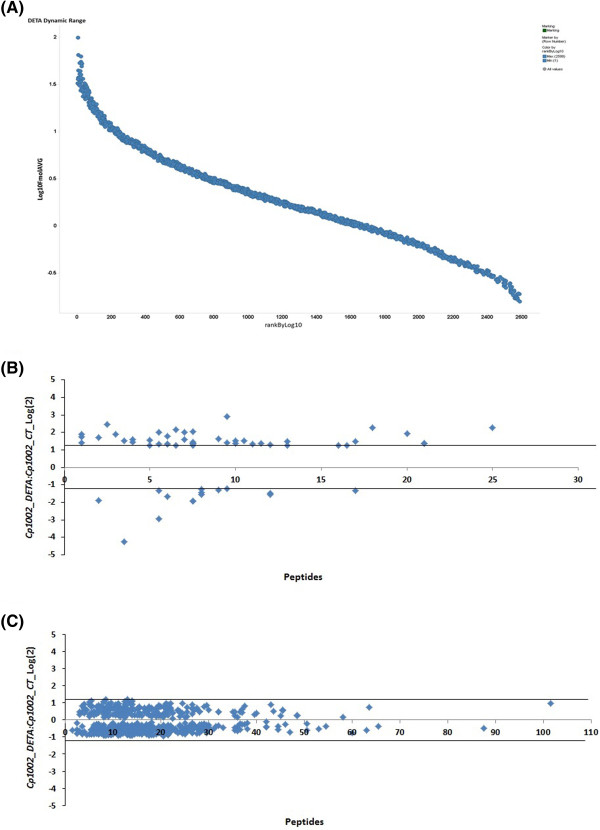
**2D nanoUPLC HDMSE analysis showing: (A) Dynamic range of the method based on protein abundance estimates, data points derived from LC-HDMS** ^**E**^ **analysis. (B and C)** Proteins that were significantly differentially-regulated during NO exposure. The distribution of identified proteins with *p* < 0.05, and differentially-regulated proteins with an I:C log_2_ ratio < 1.2 in relation to the number of peptides identified for each protein. **(B)** Proteins with *p* < 0.05 and an I:C log_2_ ratio < 1.2. **(C)** Proteins with *p* < 0.05 and an I:C log_2_ ratio > 1.2.

Total proteome digests from three biological replicates of each individual condition were subjected to LC/MS^E^. In total, we identified more than 31,000 peptides, with a normal distribution of 10 ppm error of the total identified peptides. Peptides as source fragments, peptides with a charge state of at least [M + 2H]^2+^, and the absence of decoys were factors considered to increase data quality. A combined total of 2,063 proteins were present in at least two of the three biological replicates for the two conditions tested, with an average of 15 peptides per protein, and a false discovery rate (FDR) of 0% when decoy detection was set at agreement of two out of three replicates. The proteins referred to as exclusive to one condition or another was only identified in one condition within the detection limits of the experiment (LOD). The dynamic range of the quantified proteins is about 3 logs, and proteins unique to one condition or another were only observed above the LOD of the experiment, which was determined by the sample normalization prior to injection. Therefore, in our study, all samples were normalized using “scouting runs” taking into account the stoichiometry between the intensity and molarity proportion prior to the replicate runs per condition. The dynamic range was similar for each sample, and the total amount of sample used in fmol was nearly the same. We generate a graph of protein amounts of the identified proteins from all samples against protein ranks (Figure 2A).

After, analysis by PLGS v2.5.2 software, the 2,063 proteins originally identified in two out of three replicates were narrowed down to 699 proteins with *p* ≤ 0.05. Among these proteins, 44 were up-regulated in the presence of DETA/NO, while 14 proteins were down-regulated (Table 1, Figure 2B and C). The remaining 641 proteins with *p* ≤ 0.05 and log_2_ < 1.2 that were common to the two treatments are summarized in Additional file 1. In addition to the 699 identified proteins that were present under both control and stress conditions, 34 proteins were exclusively expressed under the control conditions, and 102 proteins were exclusively expressed in response to DETA/NO stress (Additional files 2 and 3). Thus, our final list of proteins is composed of 835 proteins from *C. pseudotuberculosis*.

**Table 1:** **Proteins identified as differentially-expressed following exposure to nitrosative stress**

Uniprot access	Proteins	Score	Peptides	log _2_ DETA:CT ^(a)^	***p*** -value ^(a)^	Subcellular localization ^(c)^	Gene name	Genome ^(b)^
**Transport**								
F9Y2Z3_CORP1	Cell wall channel	5321.88	4	1.42	1	CYT	*porH*	Shared
**Cell division**								
D9Q7G2_CORP1	Hypothetical protein	2417.8	21	1.34	1	CYT	*Cp1002_0716*	Core
**DNA synthesis and repair**								
D9Q5V6_CORP1	Nucleoid-associated protein	2327.08	5	1.52	1	CYT	*ybaB*	Core
D9Q923_CORP1	Methylated-DNA-protein-cysteine methyltransferase	6332.83	8	1.22	1	CYT	*ada*	Core
D9Q4P0_CORP1	7,8-dihydro-8-oxoguanine-triphosphatase	1640.23	8	-1.97	0	CYT	*mutT*	Core
**Transcription**								
D9Q8W2_CORP1	LexA repressor	800.31	6	-1.37	0.04	CYT	*lexA*	Shared
D9Q5L4_CORP1	ECF family sigma factor k	364.82	8	-1.58	0	CYT	*sigK*	Core
**Translation**								
D9Q753_CORP1	Fkbp-type peptidyl-prolyl cis-trans isomerase	7113.34	3	2.43	1	CYT	*fkbP*	Core
D9Q830_CORP1	50S ribosomal protein L35	2271.66	1	1.36	1	CYT	*rpmI*	Core
D9Q7W1_CORP1	Aspartyl glutamyl-tRNA amidotransferase subunit C	3100.8	7	1.24	0.99	CYT	*gatC*	Core
D9Q582_CORP1	50S ribosomal protein L9	41082.46	10	-1.25	0	CYT	*rplI*	
D9Q6H6_CORP1	30S ribosomal protein S8	45333.23	9	-1.34	0	CYT	*rpsH*	Core
**Cell communication**								
D9Q559_CORP1	Hypothetical protein	1402.27	6	1.99	1	PSE	*Cp1002_2005*	Core
D9Q5U9_CORP1	Thermosensitive gluconokinase	2068.35	7	1.96	0.99	CYT	*gntK*	Core
D9Q668_CORP1	Sensory transduction protein RegX3	2540.92	13	1.45	1	CYT	*regX3*	Core
**Detoxification**								
D9Q7U6_CORP1	Thioredoxin	1835.7	11	1.50	1	CYT	*trxA*	Core
D9Q4E5_CORP1	Glutathione peroxidase	1426.27	10	1.47	1	CYT	*Cp1002_1731*	Core
D9Q5T5_CORP1	Glyoxalase bleomycin resistance protein dihydroxybiphenyl dioxygenase	2417.77	11	1.28	1	CYT	*Cp1002_0124*	Shared
D9Q5N2_CORP1	NADH dehydrogenase	7030.94	12	1.25	1	CYT	*noxC*	Shared
D9Q680_CORP1	Glutaredoxin-like domain protein	292.69	2	-1.91	0	CYT	*Cp1002_0272*	Core
**Glycolysis pathways**								
D9Q5B6_CORP1	N-Acetylglucosamine kinase	228.69	6	1.74	0.98	CYT	*nanK*	Core
D9Q4U9_CORP1	Alcohol dehydrogenase	236.02	17	1.22	1	CYT	*adhA*	Shared
**Iron-sulfur clusters**								
D9Q7L6_CORP1	Ferredoxin	36927.57	7	2.10	1	CYT	*fdxA*	Core
**Antibiotic resistance**								
D9Q827_CORP1	Metallo-beta-lactamase superfamily protein	657.33	6	-2.95	0	CYT	*Cp1002_0937*	Core
**Amino acid metabolism**								
D9Q622_CORP1	Phosphoserine phosphatase	949.15	9	1.58	0.99	PSE	*serB*	Core
D9Q4N1_CORP1	Carboxylate-amine ligase	205.54	16	1.24	1	CYT	*Cp1002_1819*	Core
D9Q6H4_CORP1	L-serine dehydratase I	284.11	17	-1.37	0	MEM	*sdaA*	Core
**Lipid metabolism**								
D9Q520_CORP1	Glycerophosphoryl diester phosphodiesterase	2417.8	21	1.34	1	PSE	*glpQ*	Core
**Oxidative phosphorylation**								
D9Q8I5_CORP1	Cytochrome aa3 controlling protein	676.2	6	1.28	1	MEM	*Cp1002_1095*	Core
**Specific metabolic pathways**								
D9Q5M9_CORP1	Inositol-3-phosphate synthase	7473.38	18	2.25	1	CYT	*ino1*	Core
D9Q721_CORP1	Hypothetical protein	4602.9	17	1.44	1	SEC	*Cp1002_0573*	Core
D9Q689_CORP1	3-Hydroxyisobutyrate dehydrogenase	2137.24	12	1.34	1	CYT	*mmsB*	Core
D9Q4X1_CORP1	Urease accessory protein UreG	1532.39	12	-1.6	0	CYT	*ureG*	Core
**Nucleotide metabolism**								
D9Q4S2_CORP1	Orotate phosphoribosyltransferase	2618.52	8	-1.26	0	CYT	*pyrE*	Core
**Unknown function**								
D9Q6Y9_CORP1	Hypothetical protein	491.89	10	2.87	1	CYT	*Cp1002_0540*	Core
D9Q6C7_CORP1	Hypothetical protein	689.6	25	2.25	1	PSE	*Cp1002_0320*	Core
D9Q3P3_CORP1	Hypothetical protein	5703.38	3	1.87	1	CYT	*Cp1002_1474*	Core
D9Q5V4_CORP1	Hypothetical protein	994.52	1	1.7	1	CYT	*Cp1002_0143*	Core
D9Q610_CORP1	Hypothetical protein	27217.36	2	1.67	1	CYT	*Cp1002_0202*	Core
D9Q8D8_CORP1	Hypothetical protein	2324.12	7	1.57	0.98	CYT	*Cp1002_1048*	Shared
D9Q6W1_CORP1	Hypothetical protein	9303.91	4	1.54	1	CYT	*Cp1002_0512*	Core
D9Q6V5_CORP1	Hypothetical protein	1346.2	4	1.5	0.99	CYT	*Cp1002_0506*	Core
D9Q5R7_CORP1	Hypothetical protein	2090.7	8	1.42	1	CYT	*Cp1002_0105*	Core
D9Q917_CORP1	Hypothetical protein	555.89	10	1.37	1	PSE	*Cp1002_1281*	Core
D9Q3P5_CORP1	Hypothetical protein	1121.7	6	1.29	1	SEC	*Cp1002_1476*	Core
D9Q7U5_CORP1	Hypothetical protein	517.06	8	1.28	1	CYT	*Cp1002_0852*	Core
D9Q7L1_CORP1	Hypothetical protein	15693.97	6	1.28	1	SEC	*Cp1002_0766*	Core
D9Q3P6_CORP1	Hypothetical protein	1729.59	5	1.22	1	CYT	*Cp1002_1477*	Core
D9Q6Z7_CORP1	Hypothetical protein	1835.7	13	1.22	1	CYT	*Cp1002_0548*	Core
D9Q8V8_CORP1	Hypothetical protein	293.23	8	-1.48	0		*Cp1002_1221*	Core
D9Q6C8_CORP1	Hypothetical protein	413.31	12	-1.52	0	PSE	*Cp1002_0321*	Core
D9Q5H0_CORP1	Hypothetical protein	12376.2	6	-1.71	0	CYT	*Cp1002_0007*	Core
D9Q4D5_CORP1	Hypothetical protein	10161.64	4	-4.29	0	CYT	*Cp1002_1721*	Shared
**Others**								
D9Q5N5_CORP1	Iron-regulated MEM protein	992.54	8	2.01	0	PSE	*piuB*	Core
D9Q922_CORP1	CobW/HypB/UreG, nucleotide-binding	1771.22	20	1.88	1	CYT	*Cp1002_1286*	Core
D9Q8C4_CORP1	Prokaryotic ubiquitin-like protein Pup	2194.86	1	1.84	1	CYT	*pup*	Core
D9Q7B8_CORP1	Ribosomal-protein-alanine n-acetyltransferase	2791.1	10	1.34	1	CYT	*rimJ*	Shared
D9Q7K9_CORP1	Arsenate reductase	5147.54	8	1.32	1	CYT	*arsC*	Core

(a) Ratio values to: strain 1002*_DETA/NO*:strain 1002*_Ct*, Log(2) Ratio > 1.5, *p* > 0.95 = up-regulation, *p* < 0.05 = down-regulation.

(b) Core-genome analysis of 15 strains of *C. pseudotuberculosis*: shared = present in two or more strains; core = present in 15 strains of *C. pseudotuberculosis.*

(c) CYT =cytoplasmic, MEM = membrane, PSE = potentially surface-exposed, SEC = secreted.

### *In silico*analysis of LC-HDMS^E^data

**Figure 3:**
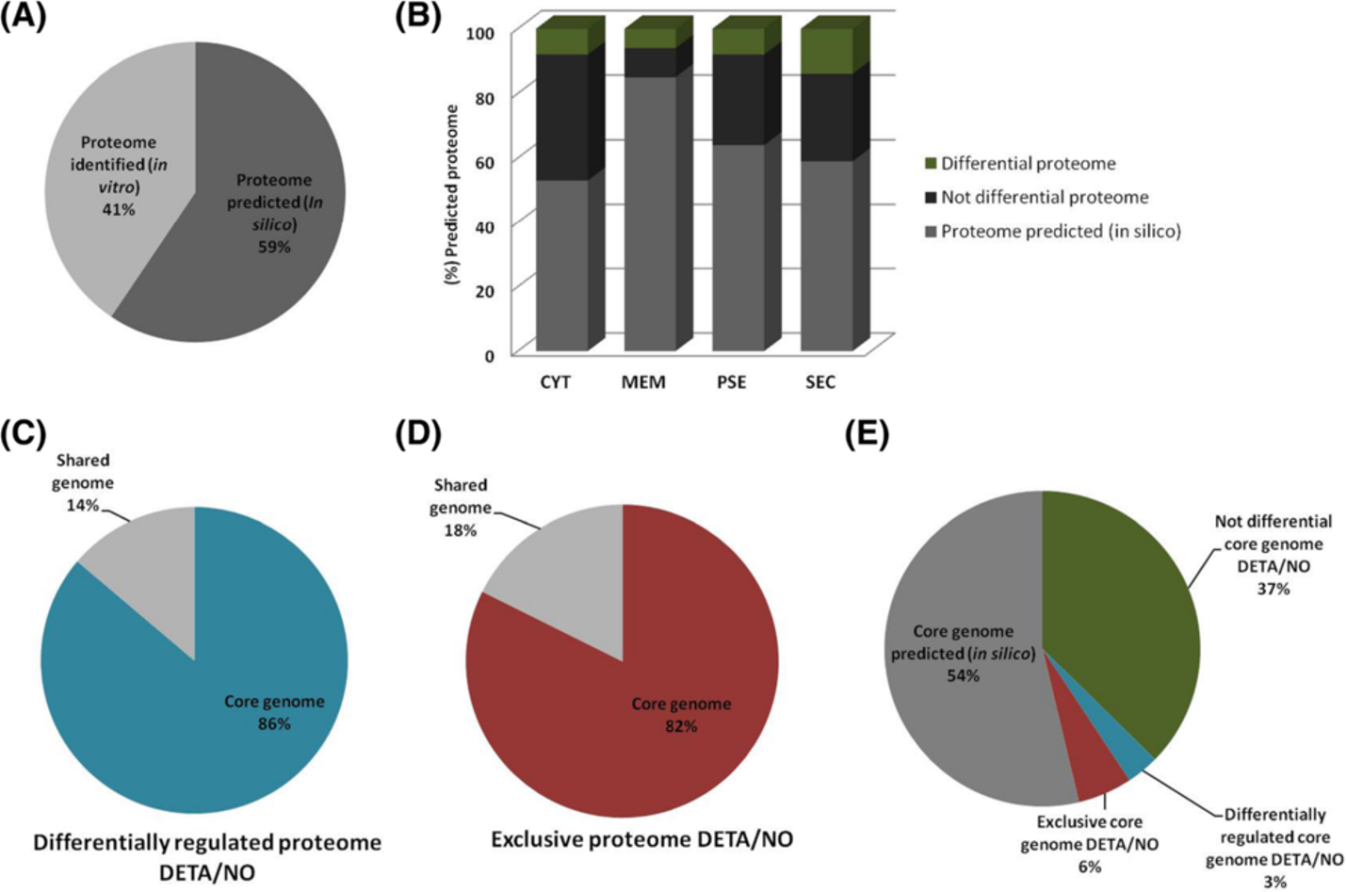
**Correlation of** ***in silico*** **predicted data with proteome results. (A)** Percentage of coverage of the *C. pseudotuberculosis* 1002 *in silico* proteome. **(B)** Prediction of the subcellular localization of the proteins identified by LC/MS. **(C)** Analysis of the differentially-regulated proteins of cells exposed to DETA/NO in relation to the core genome of *C. pseudotuberculosis* (shared genome: present in only two strains; core genome: present in 15 strains of *C. pseudotuberculosis*). **(D)** Analysis of the exclusive proteome of cells exposed to DEA/NO in relation to the core-genome of *C. pseudotuberculosis* (shared genome: present in only two strains; core genome: present in 15 strains of *C. pseudotuberculosis*). **(E)** Percent coverage of the core-genome of *C. pseudotuberculosis* in relation to the characterized proteome *in vitro*.

The 835 proteins were then analyzed using the SurfG+ tool to predict sub-cellular localization. According with SurfG+, our data set included approximately 41% of the predicted proteome of strain 1002 (Figure 3A). In addition, we characterized proteins belonging to the following cell fractions: cytoplasmic (CYT) (668 proteins), membrane (MEM) (59 proteins), potentially surface-exposed (PSE) (69 proteins), and secreted (SEC) (39 proteins) (Figure 3B).

To evaluate whether the proteins identified in our proteomic analysis could represent a protein set expressed by *C. pseudotuberculosis* during exposure to nitrosative stress, we correlated our proteomic data with the predicted core-genomes of 15 *C. pseudotuberculosis* strains [7]. Of the open reading frames (ORFs) coding for the differentially-regulated proteins and exclusive proteome of DETA/NO-exposed cells, 86% (50/58 proteins) and 82% (84/102 proteins) were identified, respectively, in the core-genome of *C. pseudotuberculosis* (Figure 3C and D). In addition, of the 835 total proteins identified from the proteome of strain 1002 following exposure to nitrosative stress, 83% (696 proteins) of the ORFs coding for these proteins were present in the core-genome of *C. pseudotuberculosis,* this result correspond approximately 46% of the predicted core-genome of *C. pseudotuberculosis* (Figure 3E).

### Functional classification of the proteome of *C. pseudotuberculosis*expressed under exposure to nitrosative stress

**Figure 4:**
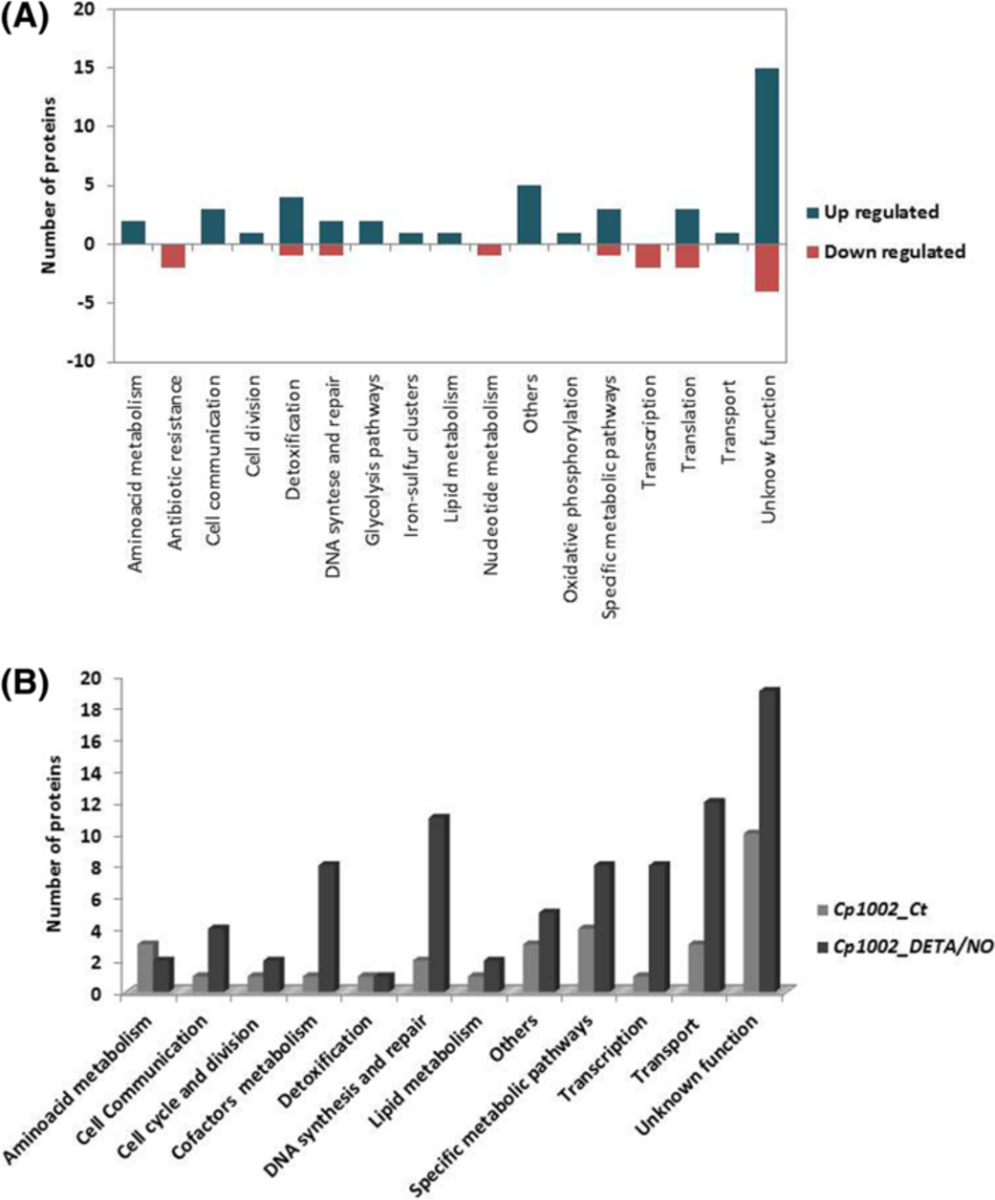
**Comparison of biological processes between control and DETA/NO conditions.** A representation of the biological processes in relation to a set list of proteins identified as **(A)** differentially-regulated in DETA/NO-stressed cells and **(B)** comparison of exclusive biological process between the two test conditions.

**Figure 5:**
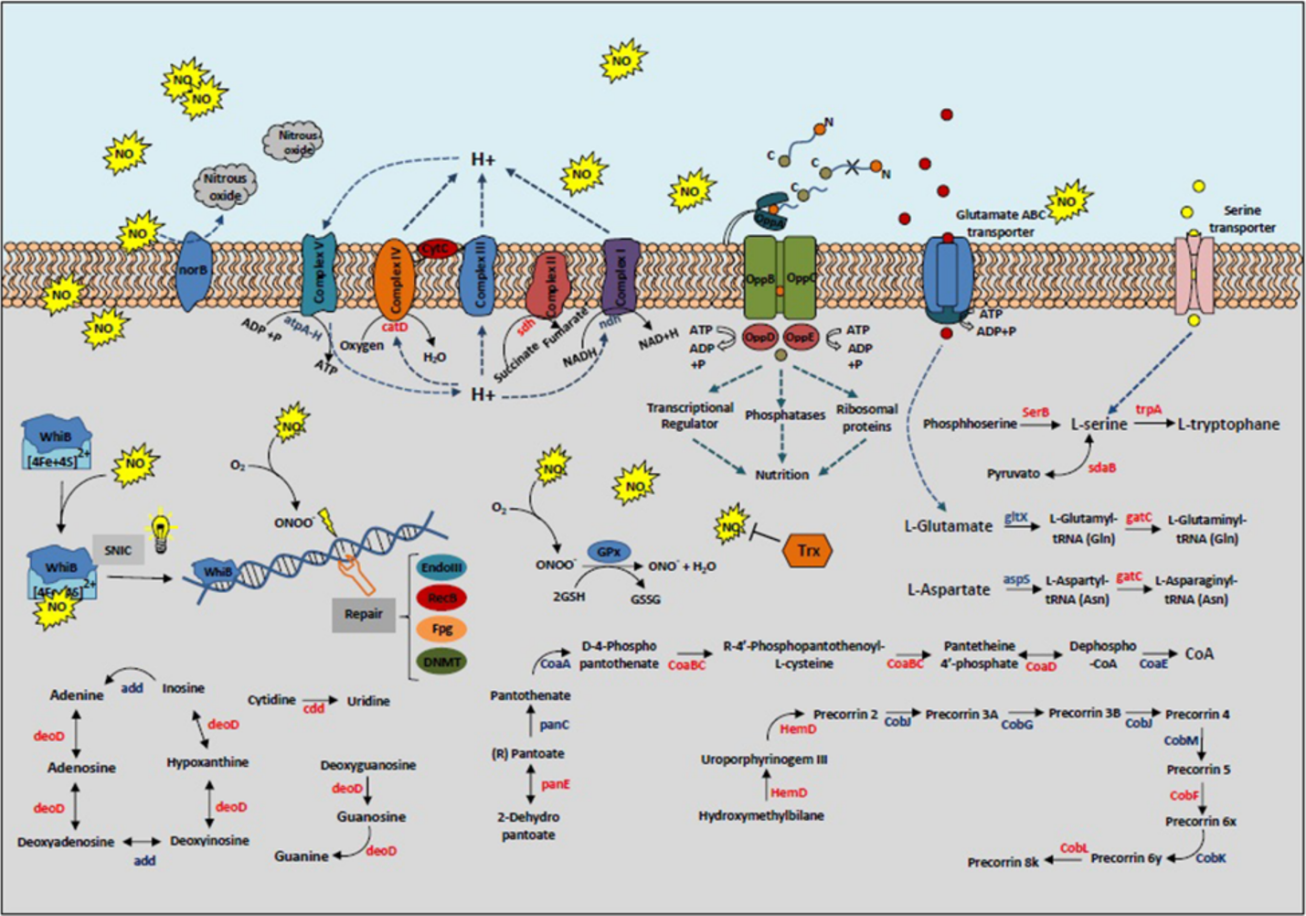
**Overview of** ***C. pseudotuberculosis*** **response to nitrosative stress.** All proteins detected by proteomic analysis are marked in red (differentially-regulated proteins or exclusive to the proteome of DETA/NO-stressed cells).

The strain 1002 proteome was functionally classified using the Blast2Go tool [24]. A large proportion of the differentially-regulated proteins and those exclusive to one condition were identified as hypothetical proteins. According to the biological function prediction, 18 biological processes were classified as differentially regulated (Figure 4A). In addition, the analysis of the exclusive proteome of each condition revealed 12 common processes between the control and stress conditions (Figure 4B). However, seven biological processes were identified only in stress-exposed cells. These processes were antibiotic metabolism (six proteins), nucleotide metabolism (five proteins), oxidative phosphorylation (three proteins), translation (three proteins), glycolysis pathways (one protein), iron-sulfur clusters (one protein), and starch and sucrose metabolism (one protein). Among all processes identified, DNA synthesis and repair proteins (14 proteins) were most common. An overview of the *C. pseudotuberculosis* response to nitrosative stress according with the proteins identified is shown in Figure 5.

The proteins that were grouped into of transcriptional process were evaluated by CMRegNet and among regulators identified; we identified the GntR- family regulatory protein (D9Q5B7_CORP1), genes regulated by GntR-type regulators are usually involved in carbohydrate metabolism. The CMRegNet analysis showed that of the four genes under the control of this regulator, the N-acetylglucosamine kinase (D9Q5B6_CORP1) protein was highly expressed by *C. pseudotuberculosis* in response to DETA/NO. We identified other regulator the LexA repressor (D9Q8W2_CORP1) that was down regulated in the DETA/NO condition. According with CMRegNet, two proteins regulated by this repressor were detected in the DETA/NO proteome specific, pyridoxal biosynthesis lyase (PdxS; D9Q5T9_CORP1) and DNA translocase (D9Q8Z6_CORP1). Others proteins under the control of this repressor was detected, however not presented significant differential regulation like RecA protein

### Protein-protein interaction network

**Figure 6:**
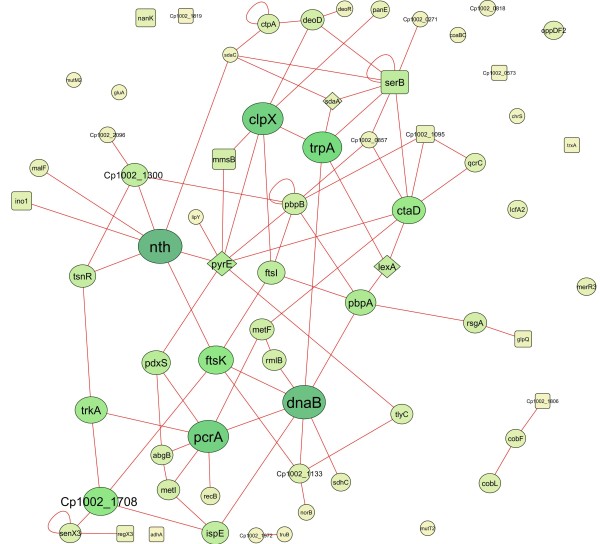
**Protein-protein interactions.** Protein-protein interactions of the proteins identified in DETA/NO-exposed cells. Exclusive proteome, circle; up-regulated, square; and down-regulated, rhombus. The sizes of the nodes represent the degree of interaction for each gene/protein; the major nodes demonstrate greater interactions. The colors of nodes and lines are in an increasing gradient scale from yellow to green to blue. The networks were visualized using Cytoscape.

To investigate the interactions among the proteins identified as exclusive and differentially regulated in cells exposed to DETA/NO, we generated a protein interaction network using Cytoscape. The interactome analysis revealed 67 protein-protein interactions (Figure 6). DnaB/DNA helicase (D9Q578_CORP1), identified in the exclusive proteome for strain 1002*_DETA/NO*, and PyrE/orotate phosphoribosyltransferase (D9Q4S2_CORP1), which was down-regulated in strain 1002*_DETA/NO*, showed the greatest number of interactions with other proteins (eight interactions each). Moreover, both of these proteins interact with proteins that are involved in metabolic processes, DNA processes, antibiotic metabolism, cell cycling, and translation.

## Discussion

*C. pseudotuberculosis* is exposed to different forms of oxidative and nitrosative stress during the infection process. A previous study showed that *C. pseudotuberculosis* resists nitrosative stress generated by the NO-donor DETA/NO, and that a low concentration of DETA/NO (100 μM) induces a change in the extracellular proteome this pathogen [15]. To better understand the physiology of *C. pseudotuberculosis* in response to nitrosative stress, we analyzed the proteome of whole bacterial lysates of *C. pseudotuberculosis* in response to exposure to DETA/NO (0.5 mM).

### The strain 1002 proteome under nitrosative stress reveals proteins involved in bacterial defense against DNA damage

Proteomic analysis identified proteins involved in DNA repair systems in both the exclusive proteome of DETA/NO-exposed cells and in the differentially-regulated proteome. We detected the proteins formamidopyrimidine-DNA glycosylase (Fpg) (D9Q598_CORP1), RecB (D9Q8C9_CORP1), and methylated-DNA-protein-cysteine methyltransferase (Ada) (D9Q923_CORP1), the genes for which were previously identified in a transcriptome analysis of strain 1002 in response to different abiotic stresses [8]. Activation of these proteins in response to nitrosative stress confirms that they belong a group of general stress-response proteins in *C. pseudotuberculosis*.

The expression of Fpg was up-regulated in response to acid stress [8]. We also identified endonuclease III (Endo III) (D9Q615_CORP1), which, in addition to Fpg, is involved in the base excision repair (BER) system of various bacteria. This system cleaves N-glycosidic bonds from damaged bases, allowing their excision and replacement. In *Salmonella enterica* serovar Typhimurium, the BER system repairs DNA damaged by exposure to NO. In addition, an *S*. Typhimurium strain defective in Fpg demonstrated reduced virulence in a murine model [30]. Our interactome analysis showed that Endo III had one of the highest numbers of interactions with other proteins, including interactions with proteins involved in DNA replication such zinc metalloprotease (D9Q378_CORP1) and DNA translocase (D9Q8Z6_CORP1), suggesting that this protein could play an important role in the defense pathway against RNS.

The Ada and RecB protein were up-regulated in response to osmotic stress [8]. Ada is involved in the repair of DNA-methylation damage, this protein have plays important in the pathway DNA damage [31]. RecB is a component of the RecBC system, which is part of the SOS response the more regulatory network encoded by prokaryotic involved in DNA repair [32]. The RecBC system acts in the recombination or degradative repair of arrested DNA replication forks. Studies in *S.* Typhimurium showed that *recBC* mutant strains are more attenuated than *recA* mutants in a murine model of infection [33]. In addition, unlike *recA* mutants, *recBC* mutants were susceptible to RNS [34], indicating that RecBC is highly important in the bacterial response to nitrosative stress. The LexA repressor (D9Q8W2_CORP1), which forms part of the general SOS system along with RecA [35], was down-regulated in *C. pseudotuberculosis* cells exposed to DETA/NO. We also detected the RecA protein (D9Q8Y3_CORP1); however, despite having a *p*-value <0.05, the fold-change of -0.50 showed that this protein was not activated under the experimental conditions. Studies performed in *Mycobacterium tuberculosis* showed that *recA* was not induced until cells had been exposed to DETA/NO (0.5 mM) for 4 h, but that hydrogen peroxide induced the immediate expression of *recA*[36], suggesting that RecA is involved in the later stages of the nitrosative stress response. Nevertheless, CMRegNet analysis identified other proteins that are regulated by LexA in the DETA/NO-specific proteome, including pyridoxal biosynthesis lyase (PdxS; D9Q5T9_CORP1) and DNA translocase (D9Q8Z6_CORP1).

### NO-sensitive transcriptional regulators are activated in the presence of NO

To activate these DNA repair systems, it is essential that bacteria can detect ROS and RNS, and concomitantly activate the transcriptional regulators needed for the expression of genes involved in protection against these compounds. In the DETA/NO-specific proteome, we detected the transcription factor WhiB (D9Q6Y2_CORP1). The WhiB transcriptional family is composed of iron-sulfur (Fe-S) cluster proteins. These proteins are O_2_- and NO-sensitive, and allow the sensing of both external environmental signals and the redox state for intracellular bacteria [37, 38]. In *M. tuberculosis,* the reaction of the iron-sulfur cluster of WhiB3 with NO generates a dinitrosyl iron complex (DNIC), which activates a sensing mechanism in response to the NO, consequently activating a system of defense against nitrosative stress [12]. In addition, other *in vivo* and *in vitro* studies have also demonstrated that WhiB regulators play a role in the adaptation and survival of *M. tuberculosis* during exposure to redox environments [12, 39–41].

We identified other regulators that are activated in response to environmental stimuli, such as a MerR-family transcriptional regulator (D9Q889_CORP1) and a LysR-type transcriptional regulator (LTTR) (D9Q7H8_CORP1). This regulator was also highly expressed in the transcriptional response of *C. pseudotuberculosis* 1002 to acid stress [8]. MerR-type regulators have been described in the detoxification of toxic metal in several pathogenic and non-pathogenic bacteria [42]. Other studies have shown that this class of regulator plays a role in bacterial resistance to oxidative and nitrosative stress [43, 44]. LTTRs are associated with the regulation of several biological processes, as well as in the adaptive response of bacteria to different types of stress [45]. In *Vibrio cholerae*, LTTRs are associated with efflux pump regulation, which contribute to antimicrobial resistance, and are involved in colonization of the human host [46]. In pathogens like *E. coli*[47], *Enterococcus faecalis*[48], *S. enterica*[49], and *Pseudomonas aeruginosa*[50], LTTRs are involved in resistance to oxidative stress.

### The detoxification pathways of *C. pseudotuberculosis*following NO exposure

Our proteomic analysis identified proteins specifically expressed by cells exposed to DETA/NO that are involved in the detoxification process. Two of these proteins were thioredoxin (*trxA*) (D9Q7U6_CORP1) and glutathione peroxidase (D9Q4E5_CORP1). The thioredoxin and glutathione systems play major roles in thiol and disulfide balance, respectively [14]. In pathogens such as *Helicobacter pylori*, *Streptococcus pyogenes*, and *M. tuberculosis*, this system is of great importance in combating the presence of ROS/RNS [36, 51, 52]*.* A glyoxalase/dioxygenase (D9Q5T5_CORP1) was identified in the differential proteome of cells exposed to DETA/NO. This protein was previously detected in the proteome of *C. pseudotuberculosis* strain 1002 in response to 0.1 mM DETA/NO [15]. The presence of this protein suggests that glyoxalase/dioxygenase plays a role in the resistance of this pathogen to nitrosative stress.

Nevertheless, unlike *P. aeruginosa*, which contains a complete denitrification pathway [53], the predicted genome of *C. pseudotuberculosis ovis* 1002 revealed a truncated denitrification pathway. However, we detected the nitric-oxide reductase cytochrome b (NorB) (D9Q5T6_CORP1) in the exclusive proteome of DETA/NO-stressed cells. *norB*, which codes for this nitric-oxide reductase, is organized into the *norCBQDEF* operon in *Paracoccus denitrificans*[54], and into the *norCBD* operon in *P. aeruginosa*[55]. The *C. pseudotuberculosis* genome was predicted to only contain *norB*. Moreover, *norB* is located in the *Cp1002PiCp12* pathogenicity island, suggesting horizontal acquisition of the gene by this pathogen. Nitric-oxide reductase is an important protein in the denitrification process of some bacteria [56]. In *P. aeruginosa*, NorB plays a role in both the growth of the pathogen in the presence of NO, and in its survival in macrophages [55]. The flavohemoglobin Hmp is involved in the NO detoxification pathway in *S.* Typhimurium, and levels of Hmp are increased approximately two-fold in macrophages [57]. Interestingly, in *N. meningitidis,* NorB levels are increased ten-fold in macrophages [58], demonstrating the great power of this protein in the detoxification process.

### Metabolic profile of *C. pseudotuberculosis*in response to nitrosative stress

In addition to the presence of proteins involved in bacterial defense and detoxification pathways, strain 1002 needs to undergo metabolic adaptation to favor bacterial survival. We observed a metabolic readjustment in this pathogen in the proteomic analysis. Of the proteins involved in central carbohydrate metabolism, we detected only phosphoglycerate mutase (D9Q533_CORP1) and N-acetylglucosamine kinase (D9Q5B6_CORP1) in the proteome of DETA/NO-exposed cells. Other essential proteins involved in glycolysis (the Embdem-Meyerhof pathway), the pentose phosphate pathway, and the citric acid cycle were not detected. Similar results were found in a metabolomic study of *V. cholerae* in response to nitrosative stress [59].

However, we hypothesized that *C. pseudotuberculosis* uses oxidative phosphorylation to obtain energy. This is supported by the presence of cytochrome C oxidase polypeptide I (D9Q486_CORP1), succinate dehydrogenase cytochrome b556 subunit (D9Q650_CORP1), and ubiquinol-cytochrome C reductase cytochrome C subunit (D9Q3J7_CORP1) in the exclusive proteome of DETA/NO-stressed cells, and by the up-regulation of the cytochrome oxidase assembly protein (D9Q8I5_CORP1) under the same conditions. However, this oxidative phosphorylation may be associated with the bacterial culture conditions used in this work, in which *C. pseudotuberculosis* was cultivated in the presence of DETA/NO under aerobic conditions. Studies have shown that growing *M. tuberculosis* in a low concentration of NO with low levels of O_2_ can induce anaerobic respiration as a result of the inhibition of the respiratory proteins cytochrome *c* oxidase and NADH reductase by irreversible ligation of NO. The ligation of NO to the respiratory proteins is an effect that may be both short-term reversible and long-term irreversible [60]. Thus, we suggest that activation of the oxidative phosphorylation system may be a more effective pathway for this pathogen to obtain energy [61].

Another metabolic adjustment was observed in relation to amino acid biosynthesis. Transporters and enzymes involved in the synthesis of methionine, tryptophan, and serine were identified. However, the presence of these proteins can be associated with the bioavailability of these amino acids during exposure to NO. In addition, we detected two oligopeptide transport ATP-binding proteins (OppD) (D9Q6G5_CORP1/ D9Q3X0_CORP1) that compose the oligopeptide permease system (Opp). This complex is associated with the internalization of peptides from the extracellular environment to be used as a source of carbon and nitrogen in bacterial nutrition [62]. We also identified proteins that are cofactors of metabolism, such as CoaBC (D9Q8L2_CORP1), phosphopantetheine adenylyltransferase (D9Q809_CORP1), and 2-dehydropantoate 2-reductase (D9Q7J9_CORP1). The presence of these proteins demonstrates activity in pantothenic acid metabolism and the biosynthesis of coenzyme A (CoA). Studies performed in species such as *Corynebacterium diphtheriae*[63], *Streptococcus haemolyticus*[64], and *M. tuberculosis*[65] showed that pantothenic acid and CoA could have an important role in the growth and viability of these pathogens.

## Conclusions

In this work, we applied high-throughput proteomics to characterize the proteome of *C. pseudotuberculosis ovis* 1002 following exposure to NO. Our proteomic analysis generated two profiles, which together validated findings from previous *in silico* analyses of *C. pseudotuberculosis ovis* 1002. The proteomic profile generated after the addition of the NO-donor, DETA/NO (0.5 mM), revealed a set of proteins that are involved in distinct biological process. We detected proteins related to both the general stress response and to a more specific nitrosative stress response, which together form a network of factors that promote the survival of this pathogen under stress conditions. However, more detailed studies are needed to assess the true role of these proteins in response to nitrosative stress in *C. pseudotuberculosis*. In conclusion, this functional analysis of the genome of *C. pseudotuberculosis* shows the versatility of this pathogen in the presence of NO. Moreover, the results presented in this study provide insights into the processes of resistance of *C. pseudotuberculosis* during exposure to nitrosative stress.

## Electronic supplementary material

Additional file 1: Table S1: Complete list of proteins identified as significantly altered (*p* < 0.05). (XLSX 169 KB)

Additional file 2: Table S2: Unique proteins identified in strain 1002*_DETA/NO*. (XLSX 21 KB)

Additional file 3: Table S3: Unique proteins identified in strain 1002 control condition. (XLSX 14 KB)

## Abbreviations

G: Guanine
C: Citosine
NO: Nitric oxide
RNS: Reactive nitrogen species
NOS: Nitric oxide synthases
LC-HDMS^E^: Liquid chromatograph high definition mass spectrometry
LC/MS: Liquid chromatograph mass spectrometry
CDM: Chemically-defined médium
DETA/NO: Diethylenetriamine/nitric oxide adduct
DTT: Dithiothreitol
2D-RP: Two-dimensional reversed phase
nanoUPLC: Nano Ultra performance liquid chromatography
nanoESI-HDMS: Nano electrospray high definition mass spectrometry
HSS: High strength silica
PLGS: Protein lynx global server
FDR: False discovery rate.
